# Geranyl Diphosphate Synthases GDS 1 and GDS7 Facilitate Natural Rubber Biosynthesis in *Taraxacum kok-saghyz* Roots

**DOI:** 10.3390/plants14192980

**Published:** 2025-09-26

**Authors:** Baoqiang Wang, Boxuan Yuan, Guoen Ao, Xiaoyou Wu, Fengyan Fang, Shiqi Long, Shugang Hui

**Affiliations:** Key Laboratory of Plant Resources Conservation and Germplasm Innovation in Moun-Tainous Region, Ministry of Education, Institute of Agro-Bioengineering, College of Life Sciences, Guizhou University, Guiyang 550025, China; bqwang0808@163.com (B.W.); yuanboxuan111@163.com (B.Y.); aoguoen@163.com (G.A.); xywu123123@163.com (X.W.); fangfengyan0913@163.com (F.F.); longshiqi77@163.com (S.L.)

**Keywords:** *Taraxacum kok-saghyz*, geranyl diphosphate synthase, transient transformation, natural rubber content, photosynthetic efficiency

## Abstract

*Taraxacum kok-saghyz* Rodin, an important rubber-producing plant, has emerged as a potential alternative crop for the natural rubber industry. Geranyl diphosphate synthase (GDS) catalyzes the condensation of dimethylallyl pyrophosphate and isopentenyl pyrophosphate into geranyl pyrophosphate in the mevalonate pathway in plants. However, its specific functions in natural rubber biosynthesis in *T. kok-saghyz* remain unclear. Methods: We conducted genome-wide analyses of *TkGDS* genes, followed by transient transformation assay, expression profiling, natural rubber quantification, and analysis of *T. kok-saghyz* photosynthesis. Results: Seven *TkGDS* genes are located on chromosomes A6 and A7 with an uneven distribution. All encoded TkGDS proteins contain FARM and SARM motifs. TkGDS1, TkGDS2, and TkGDS7 possess *lspA* domains, while TkGDS3, TkGDS4, TkGDS5, and TkGDS6 contain PLN02890 domains; both subgroups share similar domain architecture. TkGDS1, TkGDS2, and TkGDS7 exhibit interspecies collinearity with *Arabidopsis thaliana*; no intraspecies collinearity was detected. The putative cis-acting elements in promoter region of *TkGDS* genes mainly comprised abscisic acid responsiveness, anaerobic induction, light responsiveness, and MeJA responsiveness. Transient expression assays confirmed chloroplast localization of all TkGDS proteins. A strong positive correlation was observed between *TkGDS1/TkGDS7* expression and natural rubber content, as confirmed by both transcriptome and qPCR analyses in *T. kok-saghyz* lines. Furthermore, overexpression of *TkGDS1* and *TkGDS7* improved photosynthetic efficiency and significantly increased natural rubber content (*OE-TkGDS1*: 6.08 ± 0.16%; *OE-TkGDS7*: 5.62 ± 0.32%; WT: 4.76 ± 0.28%). Conclusions: Our study elucidates the role of *GDS1* and *GDS7* in promoting growth and latex content, offering a genetic strategy for enhancing rubber accumulation in *T. kok-saghyz.*

## 1. Introduction

Natural rubber, a strategically vital biopolymer, has become an indispensable raw material in various industrial sectors due to its unique properties [1]. The primary source of natural rubber, *Hevea brasiliensis*, thrives in tropical climates and has played a pivotal role in global economic development since the industrial revolution [2,3]. However, the growing global demand for natural rubber, combined with challenges such as climate change, land-use limitations, and devastating diseases including South American leaf blight (*Microcyclus ulei*) and powdery mildew (*Oidium heveae*), exposes critical vulnerabilities in the *H. brasiliensis*-based production system [1,4]. Additionally, the presence of rising labor costs further complicates its cultivation [1,5]. Collectively, these limitations underscore the urgent need to explore alternative sources of natural rubber.

*Taraxacum kok-saghyz* Rodin, a resilient perennial herbaceous plant native to the Tianshan Valley basin, has emerged as a promising alternative. This temperate-adapted plant accumulates natural rubber in its roots, reaching from about 3% in annual plants up to 28% in biennial plants of root dry weight [6,7,8]. It’s noteworthy that rubber accumulation is significantly influenced by the plant’s age, genetic background, and growing environment. To study this genetic component, distinct lines such as the high-yielding T1001 and intermediate-yielding T601 have been developed and characterized [9]. In contrast to *H. brasiliensis*, which requires tropical conditions, *T. kok-saghyz* has a shorter rubber production cycle and is suitable for cultivation in temperate regions, making it a feasible candidate for scalable rubber production [10,11]. Given the increasing global demand for natural rubber, *T. kok-saghyz* has been identified as a highly promising alternative rubber source with the potential to alleviate future supply shortages [12]. However, its path to commercialization, as noted in the same review, is contingent upon overcoming key agronomic challenges such as inconsistent rubber yield and high phenotypic variability, which are currently the focus of intensive international research efforts.

The intricate molecular mechanisms orchestrating rubber biosynthesis have been a paramount focus of natural rubber research [13]. This biosynthesis process can be divided into three stages: (1) the synthesis of acetyl-CoA, (2) its conversion into isopentenyl pyrophosphate (IPP) via the mevalonate (MVA) pathway, and (3) the *cis*-polymerization of IPP to form high-molecular-weight rubber hydrocarbons [13]. In the final stage, geranyl pyrophosphate (GPP), a key precursor for monoterpene biosynthesis, is synthesized from IPP and dimethylallyl pyrophosphate (DMAPP) by geranyl diphosphate synthase (GDS) within plastids [14]. GDS functions either as a homodimeric or heterodimeric enzyme complex and plays a pivotal role in monoterpenes production, which is essential for rubber biosynthesis [15]. Several enzymes, including GDS, farnesyl diphosphate synthase (FPS), and geranylgeranyl diphosphate synthase (GGPPS), cooperatively regulate the formation of high-molecular-weight rubber hydrocarbons [16,17,18]. The MVA pathway connects primary and secondary metabolism by generating universal precursors like GPP, which serve as building blocks for key hormones (e.g., abscisic acid, gibberellins, carotenoids) and specialized metabolites such as strigolactones [19,20]. Its regulation is highly responsive to environmental and developmental signals, ensuring resource allocation toward monoterpene biosynthesis and allowing plants to adapt to environmental stresses [1,21].

The critical role of GDS in controlling flux into the terpenoid backbone has been functionally validated in diverse plant species through genetic and biochemical studies [22,23]. For instance, GDS is primarily localized in the chloroplasts of photosynthetically active parenchyma cells and the non-green plastids of secretory cells, which serve as major sites for monoterpene biosynthesis [24]. In *H. brasiliensis*, functional analyses have shown that GDS functions as homodimers or heterodimers, with gene expression levels positively correlated with both dry rubber content and the duration of methyl jasmonate (MeJA) treatment [22]. The enzyme’s product specificity is often determined by its subunit composition; it consists of a large catalytic subunit and a small regulatory subunit, with the latter being a key determinant of product specificity and metabolic regulation [23,25]. Structural variations in GDS subunits in gymnosperms enhance the production of specific monoterpenes in response to biotic stress [26,27]. Perhaps the most compelling functional evidence comes from gene manipulation experiments. In angiosperms such as Lycopersicon esculentum (tomato), gene silencing of *GDS* leads to reduced gibberellin levels and severe dwarfism, providing direct genetic evidence for its essential role in diterpene biosynthesis [1]. Likewise, GDS has also been implicated in abscisic acid biosynthesis, underscoring its broader influence on plant growth and development [28]. Beyond these model systems, *GDS* genes have been functionally characterized in a variety of aromatic and medicinal species, including *Mentha canadensis*, *Pogostemon cablin*, and *Paeonia lactiflora*, where they have been shown to regulate the biosynthesis of specific, economically important monoterpenes [29,30,31]. This body of work across species establishes GDS as a master regulator of terpenoid diversity and provides a strong rationale for investigating its role in the rubber biosynthesis pathway of *T. kok-saghyz*.

Despite the established role of GDS in terpenoid biosynthesis across plant species, the specific functions, regulatory mechanisms, and contributions of the *GDS* gene family to rubber biosynthesis in *T. kok-saghyz* remain largely unexplored. Key questions persist: which TkGDS isoforms are functionally involved in rubber production? How is their expression regulated in relation to natural rubber accumulation? And do they influence yield solely through precursor supply or also by modulating broader physiological processes like photosynthesis? To address these gaps, we conducted a comprehensive study combining genome-wide identification, phylogenetic analysis, expression profiling, and functional characterization. We specifically utilized two distinct *T. kok-saghyz* lines with contrasting rubber content to unravel the relationship between *TkGDS* expression and rubber yield. This study aims to pinpoint the key *TkGDS* members responsible for rubber biosynthesis and to elucidate their potential role in coordinating metabolic flux for rubber production in *T. kok-saghyz*.

## 2. Results

### 2.1. Identification of TkGDS and Phylogenetic Analysis

We identified seven geranyl diphosphate synthase gene members in *T. kok-saghyz* (Table 1). Biochemical analysis revealed that these TkGDS proteins exhibit common characteristics, including amino acid lengths ranging from 264 (TkGDS2) to 419 (TkGDS6) residues, predicted isoelectric points between 5.14 (TkGDS1) and 6.33 (TkGDS4), and molecular weights varying from 28.53 kDa (TkGDS2) to 46.38 kDa (TkGDS4). Phylogenetic analysis categorized GDS proteins into three distinct clades. The TkGDS proteins were distributed between clade II and clade III, while clade I exclusively contained Eucommia ulmoides EuGDS3. Within clade II, TkGDS1, TkGDS2 and TkGDS7 formed a cluster closely related to HbGDS1, HbGDS2, HbGDS3 and HbGDS4.

**Figure 1:**
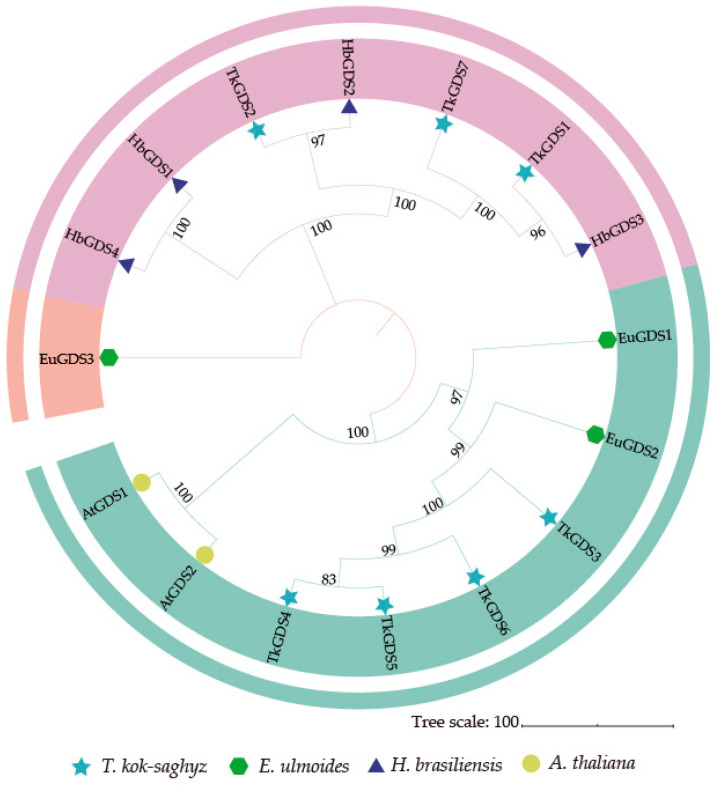
Phylogenetic and evolutionary tree of GDS proteins in four species. The evolutionary tree was constructed using the Neighbor-Joining (NJ) method. Bootstrap values greater than 50% are shown at the nodes. *T. kok-saghyz* (Tk, star), *E. ulmoides* (Eu, hexagon), *H. brasiliensis* (Hb, triangle), *A. thaliana* (At, circle) are marked with different symbols. Colors indicate different clades.

**Figure 2:**
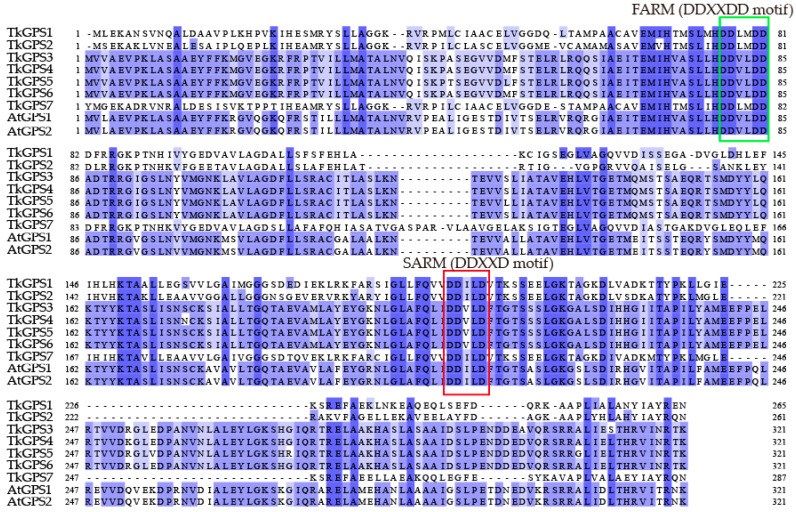
Multiple sequences alignment of GDS proteins in *A. thaliana* and *T. kok-saghyz*. Dark blue regions represent the similarities were 100%, blue regions: 67–78%, light blue ones: 45–56%, Green box: FARM, Red box: SARM.

The remaining members, TkGDS3, TkGDS4, TkGDS5 and TkGDS6, were grouped in clade III and showed evolutionary proximity to AtGDS1, AtGDS2, EuGDS1, and EuGDS2 (Figure 1). Sequence alignment with AtGDS homologs confirmed the conserved presence of both FARM and SARM (Figure 2). Collectively, these results highlight the structural conservation and evolutionary significance of the TkGDS family.

### 2.2. GDS Protein Motifs, Domains, Gene Structures Analysis and Syntenic Profiling

**Figure 3:**
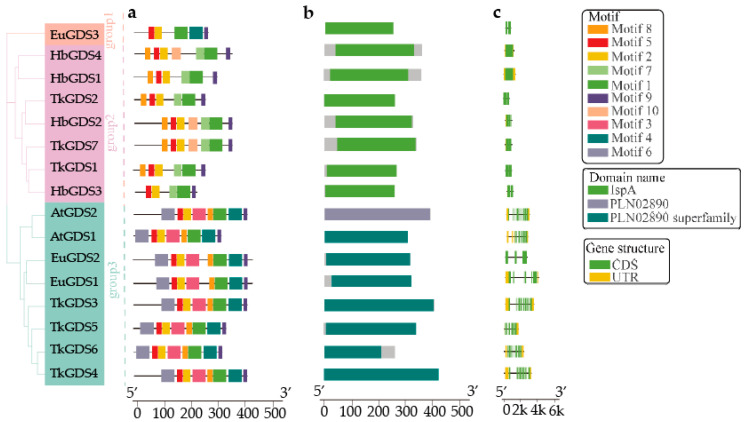
Motif, domain, CDS and UTR distributions of GDS in four species, (**a**) Motif distribution, colored bars represent distinct motifs. (**b**) conserved domain distribution, colors denote domain types. (**c**) CDS and UTR distributions, green rectangles: coding sequences (CDS), yellow rectangles: untranslated regions (UTR). Scale bars indicate sequence length.

We conducted a comparative molecular analysis of GDS proteins from *A. thaliana*, *E. ulmoides*, *H. brasiliensis* and *T. kok-saghyz*. A total of 10 conserved motifs were identified across all TkGDS proteins (Figure 3). TkGDS1, TkGDS2, and TkGDS7 formed a distinct cluster, characterized by a unique motif combination of motif 8, motif 5, motif 2, motif 7, motif 1, motif 9, and motif 10. In contrast, TkGDS3, TkGDS4, TkGDS5, and TkGDS6 shared a conserved motif pattern including motif 6, motif 5, motif 2, motif 3, motif 8, motif 1, motif 4, and motif 9 with AtGDS homologs (Figure 3a). Notably, TkGDS1, TkGDS2, and TkGDS7 contain the IspA domain (isopentenyl pyrophosphate transferase), which is essential for IPP biosynthesis. In contrast, TkGDS3, TkGDS4, TkGDS5 and TkGDS6 classified under the PLN02890 family, and lack this catalytic domain (Figure 3b). Chromosomal localization analysis revealed distinct genomic distributions. *TkGDS1*, *TkGDS2* and *TkGDS7* were located on chromosome A6, while *TkGDS3*, *TkGDS4*, *TkGDS5*, and *TkGDS6* were positioned on chromosome A7 (Figure S1).

Examination of CDS and untranslated region (UTR) structures revealed irregular genomic arrangements among *TkGDS* genes (Figure 3c and Figure S2a), Although conclusive evidence for specific duplication mechanisms is currently constrained by the available genomic resources, the absence of collinearity among *TkGDS* genes themselves suggests that gene family expansion in *T. kok-saghyz* could have potentially involved segmental duplication events. Moreover, we identified several species-specific tandem duplications in *T. kok-saghyz* that were absent in *A. thaliana*, which may indicate a possible mechanism for rapid gene family expansion through neo-functionalization under selective pressures. The limited genomic collinearity between *T. kok-saghyz* and *A. thaliana* (Figure S2b) is consistent with the notion that lineage-specific genomic rearrangements, in addition to conserved evolutionary trajectories, may have contributed to diversification within the *TkGDS* gene family.

### 2.3. Secondary and Tertiary Structural Analysis of TkGDS Proteins and Interaction Network

Secondary structure predictions revealed that α-helices were the predominant structural elements in TkGDS proteins, followed by random coils, extended strands and β turns (Table 2). The α-helical content ranged from 45.64% to 66.67%, while β-turns from 3.67% to 7.09%, extended strands from 4.35% to 12.39%, and random coils from 23.77% to 38.30%, respectively. Three-dimensional homology modeling exhibited highly reliable structures, with GMQE scores ≥ 0.84 and sequence identity to templates ranging from 71.67% to 92.42%. Structural comparisons revealed that TkGDS7 contained a unique random coil structure (Figure S3), TkGDS1, TkGDS2, and TkGDS7 shared similar overall conformation, whereas TkGDS3, TkGDS4, TkGDS5, and TkGDS6 formed a separate cluster with closely related structural features. The prevalence of α-helical domains is consistent with a putative in maintaining structural stability, while the variable random coil region might contribute to structural flexibility that facilitates functional divergence among different TkGDS members. Protein–protein interaction network analysis further demonstrated strong physical associations among TkGDS proteins (Figure S4). The network, constructed using the STRING database, showed that these interactions were supported by multiple lines of evidence, including experimentally determined data, co-expression, and protein homology. This robust predicted interactome suggests that TkGDS enzymes may function cooperatively, potentially forming homo- or heteromeric complexes to regulate metabolic flux in the rubber biosynthetic pathway.

### 2.4. Cis-Acting Elements Analysis of the Promoter of TkGDS

**Figure 4:**
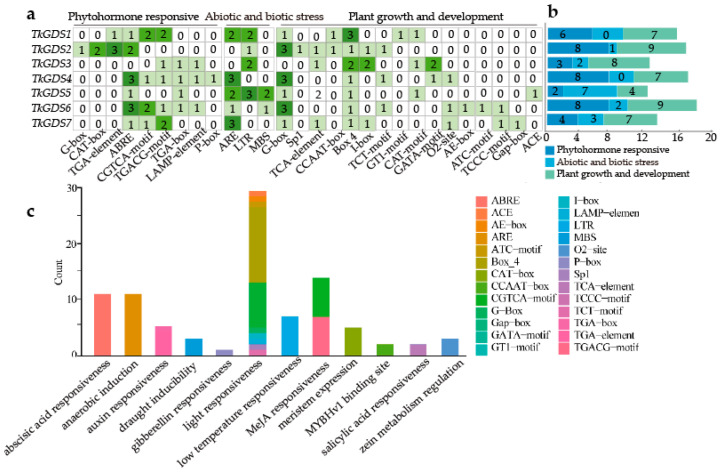
*Cis*-acting regulatory elements prediction. (**a**) Analysis of *TkGDS* genes upstream promoter cis-acting regulatory elements. Numbers in boxes represent the amounts of elements. (**b**) Total amounts of elements of different responding functions in *TkGDS* genes. (**c**) Distribution of all detected *cis*-acting regulatory elements.

A total of 26 *cis*-regulatory elements were identified in the promoter regions of *TkGDS* genes and categorized into three major groups, growth- and development-related elements, phytohormone-related elements, and stress-response-related elements (Figure 4a,b). The most prominent regulatory elements were associated with abscisic acid responsiveness, anaerobic induction, light responsiveness, and MeJA responsiveness (Figure 4c). The distribution of these elements varied among the *TkGDS* genes, indicating potential functional divergence. Auxin-responsive elements were specifically detected in *TkGDS3*, *TkGDS4*, *TkGDS5*, and *TkGDS6*, suggesting a role in development processes mediated by the auxin signaling pathway. MeJA-responsive elements were present in the promoters of *TkGDS* genes except *TkGDS2* and *TkGDS5*, suggesting that most members are likely regulated by MeJA through phytohormone signaling. The widespread presence of auxin- and MeJA-responsive regulatory elements underscores the regulatory significance of *TkGDS* genes and highlights the need for further functional investigations.

### 2.5. Subcellular Localization of TkGDS

**Figure 5:**
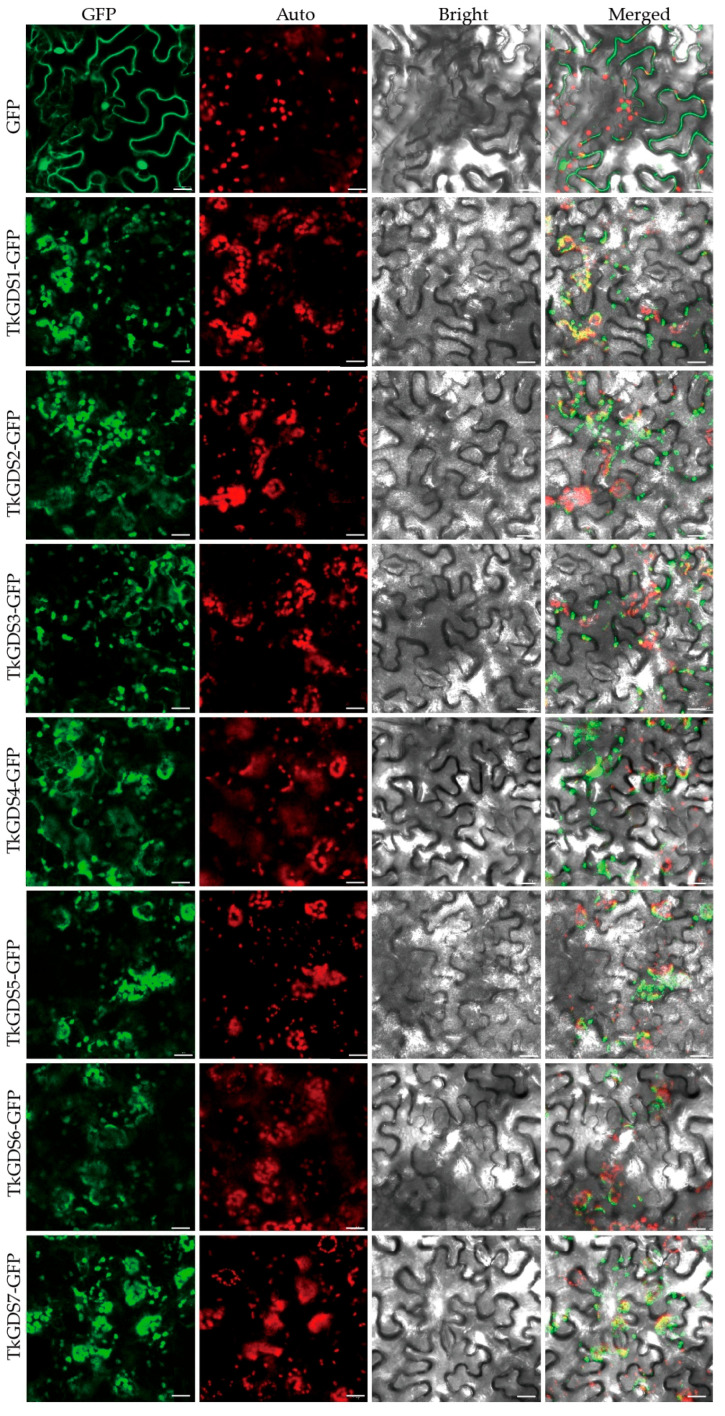
Subcellular location. GFP: 35S::GFP. The GFP fluorescence signal is shown in green, and chlorophyll auto-fluorescence is shown in red. Bright images confirm the intactness of the protoplasts. An overlay of these three is also shown (Merged). Bars = 20 μm.

To determine the subcellular localization of TkGDS proteins, we conducted subcellular localization assays using GFP fusion constructs (Figure 5). The control 35S::GFP fusion proteins exhibited strong green fluorescence in both the nucleus and plasma membrane, confirming diffuse subcellular distribution. In contrast, the GFP signals from TkGDS-GFP fusion proteins colocalized with the red autofluorescence of chloroplasts, indicating specific chloroplast targeting. No green fluorescence was detected in the nucleus or plasma membrane for TkGDS-GFP, reinforcing their organelle-specific localization. These observations demonstrated that TkGDS proteins are exclusively localized to chloroplasts, suggesting this organelle as their primary site of function.

### 2.6. Potential Role of GDS1 and GDS7 in the Regulatory Network of T. kok-saghyz

**Figure 6:**
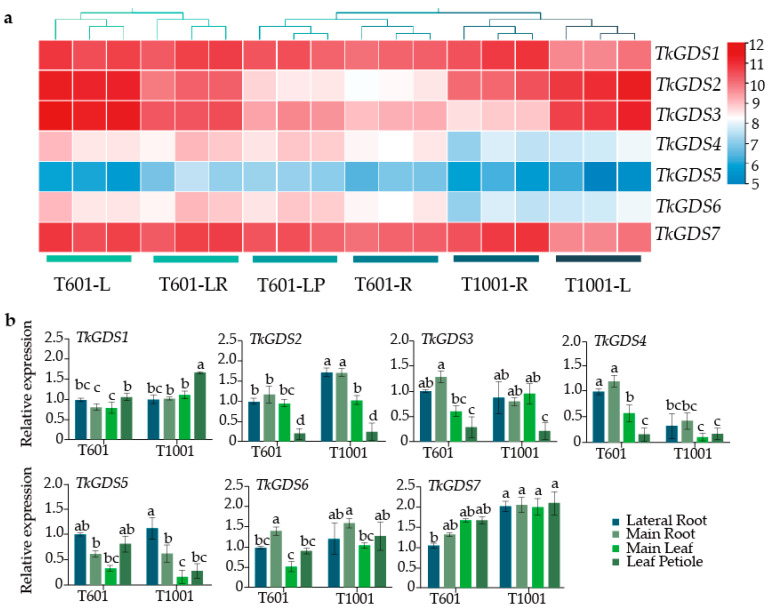
Expression patterns of *TkGDS* genes in tissues and transgenic lines. (**a**) Transcriptome analysis: L = leaf, R = root, LR = lateral root, LP = leaf petiole. (**b**) qPCR validation: WT = wild type. Bars represent mean ± SD (*n* = 3 biological replicates). Lowercase letters (a, b, c, d) indicate statistically significant differences between groups (*p* < 0.05). Groups labeled with different letters differ significantly, while identical letters indicate no significant difference.

Transcriptome analysis revealed distinct expression patterns among *TkGDS* gene family members in the T601 and T1001 lines. *TkGDS2* and *TkGDS3* showed notably high expression in leaf tissues across both lines, where *TkGDS4*, *TkGDS5*, and *TkGDS6* exhibited consistently low expression levels in all four examined tissues. In contrast, *TkGDS1* and *TkGDS7* maintained high transcript abundance in all tested tissues compared with the profile of other genes (Figure 6a), without evident line- or tissue-specific expression differences (Table S5). This stable expression across developmental stages and genetic backgrounds suggests that *TkGDS1* and *TkGDS7* may play essential, non-redundant roles in core metabolic processes crucial to plant physiology.

To further assess gene expression differences between T601 and T1001 lines, qRT-PCR was performed. The results demonstrated that *TkGDS1*, *TkGDS2* and *TkGDS7* were significantly upregulated in the main roots of the high-rubber-content T1001 line compared to T601. In contrast, *TkGDS3*, *TkGDS4*, *TkGDS5*, and *TkGDS6* maintained similar tissue-specific expression profiles across both lines. Notably, *TkGDS1* and *TkGDS7* expression levels were substantially higher in the high-rubber T1001 line than in the low-rubber T601 line in both roots and leaves, whereas *TkGDS2* showed no such difference (Figure 6b). Variance analysis indicated that *TkGDS1* expression was significantly affected by both line and tissue type (Table S6), while *TkGDS7* expression was primarily influenced by line alone (Table S7). These findings suggest that different regulatory mechanisms govern the expression of *TkGDS* genes involved in rubber biosynthesis. While *TkGDS3*, *TkGDS4*, *TkGDS5*, and *TkGDS6* showed stable, root-preferential expression independent of genotype or environment, *TkGDS1* and *TkGDS7* demonstrated line-dependent expression variation. The line-specific variation in their expression between T601 and T1001 strongly implicates them in the regulation of natural rubber biosynthesis pathways responsive to genetic and environmental factors.

### 2.7. Functional Characterization of GDS1 and GDS7 Overexpression Lines in T. kok-saghyz

**Figure 7:**
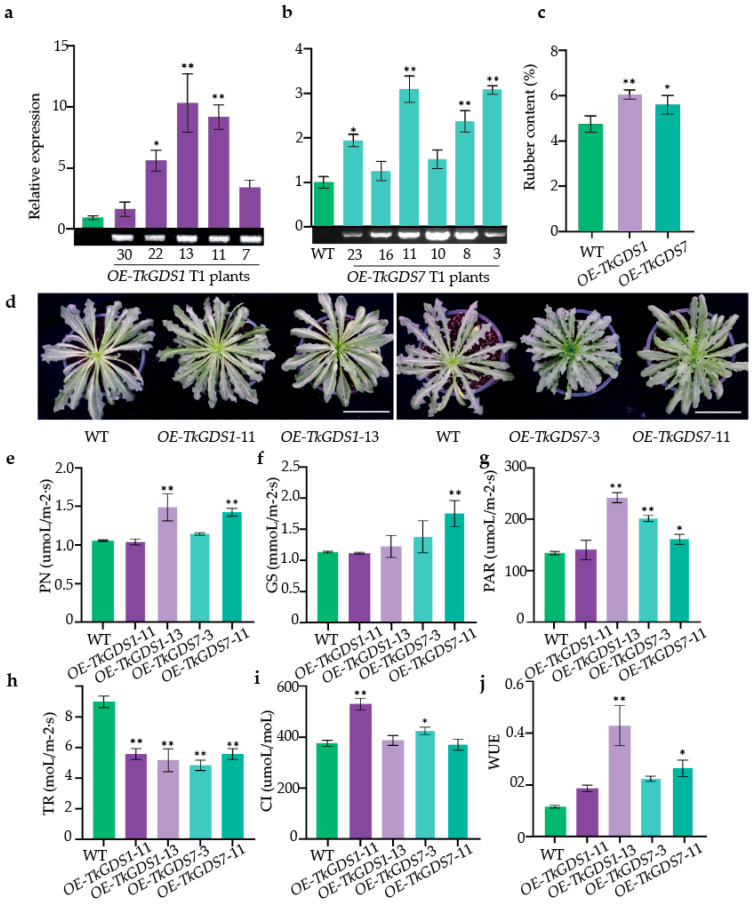
Overexpression materials analysis. (**a**) Relative expression of *OE-TkGDS1* plant materials. (**b**) Relative expression of *OE-TkGDS7* plant materials. (**c**) Rubber content of T1 plants. (**d**) Phenotypic characterization of wild-type and over-expression lines in *T. kok-saghyz*. (**e**) Net Photosynthetic Rate. (**f**) Stomatal Conductance. (**g**) Photosynthetically Active Radiation. (**h**) Transpiration Rate. (**i**) Intercellular CO_2_ Concentration. (**j**) Water Use Efficiency. *OE-TkGDS1*/*OE-TkGDS7*: transgenic lines. * Indicates *p* < 0.05, ** indicates *p* < 0.01. Data are presented as mean ± SD (*n* = 3). Significant differences were determined by one-way ANOVA and are indicated with asterisks (* *p* < 0.05, ** *p* < 0.01).

To explore how *TkGDS1* and *TkGDS7* contribute to natural rubber biosynthesis, we generated *T. kok-saghyz* transgenic lines overexpressing each gene (Figure S5). qRT-PCR analysis confirmed significantly elevated transcript levels in the overexpression lines compared to wild-type plants (Figure 7a,b; Tables S8 and S9). Specifically, *TkGDS1* expression increased by 9.21-, 10.33-, and 5.64-fold in lines *OE-TkGDS1*-11, *OE-TkGDS1*-13, and *OE-TkGDS1*-22, respectively. Likewise, *TkGDS7* expression was elevated by 3.08-, 2.36-, 3.09-, and 1.93-fold in lines *OE-TkGDS7*-3, *OE-TkGDS7*-8, *OE-TkGDS7*-11, and *OE-TkGDS7*-23. Both *OE-TkGDS1* and *OE-TkGDS7* lines exhibited moderately increased rubber accumulation compared to wild-type plants, with rubber content rising from 4.76 ± 0.28% (WT) to 5.62 ± 0.32% in *OE-TkGDS7* lines and 6.08 ± 0.16% in *OE-TkGDS1* lines (Figure 7c). Despite these increases, no notable differences in leaf morphology were observed between transgenic and wild-type plants (Figure 7d). However, physiological measures revealed that *OE-TkGDS1*-13 and *OE-TkGDS7*-11 lines demonstrated enhanced photosynthetic capacity relative to wild type and other transgenic lines. These improvements were reflected in significantly higher values of key parameters including photosynthetically active radiation (PAR) increased by 79.1% and 20.1%, net photosynthetic rate (Pn) by 41.3% and 35.0%, and intercellular CO_2_ concentration (Ci) by 3.2% and −1.2% in *OE-TkGDS1*-13 and *OE-TkGDS7*-11, respectively, compared with wild-type plants (Figure 7e,g,i). Additionally, the *OE-TkGDS1* and *OE-TkGDS7* lines exhibited improved water-use efficiency (WUE) and lower transpiration rates (TR), with 264%, 129% higher WUE and 42.4%, 38.0% lower TR, compared with wild-type plants (Figure 7j,h), respectively. Taken together, these results indicate that *TkGDS1* and *TkGDS7* may enhance natural rubber biosynthesis indirectly by improving photosynthetic performance and resource use efficiency, thereby promoting biomass accumulation and metabolic flux toward rubber production.

## 3. Discussion

In this study, we conducted a comprehensive analysis of *GDS* genes in *T. kok-saghyz* to elucidate their roles in photosynthesis and natural rubber biosynthesis. Among them, *TkGDS1* and *TkGDS7* emerged as key candidates, as their overexpression resulted in a notable increase in rubber content, suggesting their potential involvement in rubber production pathways. Phylogenetic and structural analyses revealed that TkGDS1, TkGDS2, and TkGDS7 cluster closely with homologous GDS proteins from *H. brasiliensis* and *A. thaliana*, indicating putative functional relevance across rubber-producing species. This close phylogenetic relationship suggests that the rubber biosynthesis function of these *GDS* genes is likely a result of divergent evolution from a common ancestral gene, rather than convergent evolution under similar selection pressures in latex-producing plants. Overexpression of *GDS1* and *GDS7* in *T. kok-saghyz* significantly enhanced natural rubber content and photosynthetic efficiency, as evidenced by elevated PAR, PN, and CI values, as well as improved water retention (lower TR and higher WUE). These findings support a model in which *TkGDS1* and *TkGDS7* overexpression concurrently enhances natural rubber biosynthesis and photosynthetic performance. This coordinated enhancement of both carbon assimilation and rubber biosynthesis processes suggests a role in improving overall carbon use efficiency for isoprenoid production.

Genome-wide profiling of the *TkGDS* family revealed diverse structural and regulatory features consistent with their functional divergence. Phylogenetic comparisons showed distinct similarity profiles to *GDS* genes from *H. brasiliensis* and *E. ulmoides*, suggesting potential functional diversification rubber biosynthetic capacity [32]. The presence of highly conserved motifs such as FARM and SARM, essential features of GDS proteins, is consistent with essential catalytic functions, while variable flanking regions (e.g., TkGDS7 random coil) could facilitate functional diversification [16]. Notably, TkGDS1, TkGDS2, and TkGDS7 encode the IspA domain, which is crucial for synthesizing isoprenoid precursors [33]. In contrast, TkGDS3, TkGDS4, TkGDS5, and TkGDS6 belong to the PLN02890 superfamil. The predicted secondary structures, dominated by α-helices, are consistent with the canonical fold of prenyltransferases. The unique random coil conformation observed in TkGDS7 may represent a structural feature that could influence its functional properties, potentially contributing to the functional divergence observed within the TkGDS family. Although the current data suggest that segmental duplications may have contributed to the expansion of the *TkGDS* family, future studies with chromosome-level genome assemblies will be essential to conclusively determine the genomic mechanisms underlying duplication events. Structurally homologous to geranylgeranyl diphosphate synthases (GGDS) found in *Brassica oleracea*, where they have been implicated in crop trait improvement [34]. Secondary structural analysis indicated that α-helices dominate the TkGDS proteins, with TkGDS7 displaying a unique random coil conformation. The high α-helical content across TkGDS members is a feature commonly associated with structural stability in enzymes; however, this remains a computational prediction in the present study and warrants further experimental validation through approaches such as mutagenesis or biochemical stability assays. Structural similarities among TkGDS1, TkGDS2, and TkGDS7 provide computational evidence suggesting these features could promote protein–protein interactions and enzymatic efficiency [35]. Protein interaction predictions further support the possibility of cooperative functions that may involve homo- or heterodimeric complexes.

Promoter analysis identified distinct cis-regulatory elements across *TkGDS* genes. Auxin-responsive elements were predominantly found in *TkGDS3*, *TkGDS4*, *TkGDS5*, and *TkGDS6*, hinting at a potential role in root development, as reported in model plants like *A. thaliana* [33,36]. MeJA-responsive elements were detected in all members except *TkGDS2* and *TkGDS5*, indicating differential jasmonate-mediated transcriptional regulation across the gene family.

Expression pattern analysis and subcellular localization offered further functional insights. All TkGDS proteins are localized to the chloroplasts, aligning with the known role of the MEP pathway in isoprenoid precursor production within this organelle [27,32]. This supports the idea of a dual contribution of *TkGDS* genes to both photosynthesis and rubber biosynthesis. Notably, *TkGDS1* and *TkGDS7* maintained consistently high expression across multiple tissues and lines, pointing to constitutive roles in plant metabolism. In contrast, *TkGDS2*, *TkGDS3*, *TkGDS4*, *TkGDS5*, and *TkGDS6* exhibited stable but lower expression levels, regardless of tissue type or line. qRT-PCR data revealed that *TkGDS1* and *TkGDS7* were more highly expressed in the main roots of T1001 compared to T601, consistent with line-specific regulatory differences. The consistently higher expression of *TkGDS1* and *TkGDS7* in the high-rubber T1001 line compared to the low-rubber T601 line further supports their involvement in rubber biosynthesis. Variance analysis indicated that *TkGDS1* expression is influenced by both genetic background and tissue type, while *TkGDS7* is predominantly regulated by genetic background. Collectively, these results are consistent with a model in which the expression of *TkGDS1* and *TkGDS7* is influenced by genetic background and may contribute to the observed variation in rubber biosynthesis between these specific lines.

The significant phenotypic improvements observed in overexpression lines highlight the practical potential of *TkGDS1* and *TkGDS7*. The concurrent increase in rubber yield and water-use efficiency positions these genes as highly relevant targets for the genetic improvement of *T. kok-saghyz*, offering a viable strategy for developing resilient rubber crops for temperate agriculture.

Variance analysis indicated that *TkGDS1* expression is influenced by both lines and tissue type, while *TkGDS7* is predominantly regulated by genetic line. Collectively, these results suggest that *TkGDS1* and *TkGDS7* mediate rubber biosynthesis through line-dependent expression modulation in response to genetic and environmental cues. While the genetic, phylogenetic, and phenotypic evidence presented above strongly suggests that TkGDS1 and TkGDS7 function as geranyl diphosphate synthases, a key limitation of this study is the absence of direct in vitro enzymatic validation. Specifically, future work should include purifying the recombinant TkGDS1 and TkGDS7 proteins to conclusively demonstrate their ability to catalyze the condensation of DMAPP and IPP into GPP. Therefore, the assignment of direct enzymatic function remains inferred from correlative evidence. Nonetheless, the strong and consistent phenotypic response observed in the overexpression lines, increased rubber content coupled with enhanced photosynthetic parameters, which provides genetic support for their critical role in regulating rubber biosynthetic flux. Additionally, the use of wild-type controls for the *A. rhizogenes* K599-mediated overexpression experiment is a limitation, this approach is an established practice for functional screening in non-model plants [37,38]. Critically, the consistent rubber enhancement observed across transgenic lines, each verified to possess the *TkGDS* transgene and to exhibit varying degrees of transgene expression, providing evidence that the effects are linked to the introduction and expression of the *TkGDS* transgene. While the empty vector controls and a formal dose–response analysis across lines are needed in future work, the genotype-phenotype co-segregation observed here strongly argues against the phenotype being solely a non-specific consequence of the Ri T-DNA background.

Furthermore, while our study primarily relies on transcriptional data, the significant increase in rubber content in *TkGDS1* and *TkGDS7*-overexpressing lines provides strong phenotypic evidence for the functional presence and activity of the encoded enzymes. This approach is well-supported in the literature on plant metabolic engineering, where a strong correlation between transgene overexpression, and desired product accumulation is often accepted as a reliable indicator of successful protein function, particularly when robust phenotypic changes are observed [39,40]. In the context of rubber biosynthesis, the overexpression of key pathway enzymes, such as small rubber particle protein 3 (SRPP3) has been repeatedly shown to directly increase rubber yield in *T. kok-saghyz*, with the measurable end-product accumulation serving as the primary validation of successful engineering [41]. Although we cannot rule out potential post-transcriptional regulation, the consistent and substantial phenotypic outcome we observed across multiple independent transgenic lines strongly suggests that the overexpressed *TkGDS1* and *TkGDS7* transcripts were successfully translated into functional proteins that catalyzed increased GPP production, thereby enhancing the flux toward natural rubber biosynthesis.

Taken together, our results support the model that *GDS1* and *GDS7* act as important regulators in coordinating photosynthetic efficiency and rubber biosynthesis in *T. kok-saghyz*. Their ability to be associated with enhanced chloroplast functionality and increased metabolic flux toward rubber precursor synthesis positions them as prime targets for improving rubber yield through genetic engineering. We propose that these genes contribute to rubber production by potentially improving the carbon and energy availability needed for synthesis, offering a valuable strategy for sustainable rubber production in alternative crop species.

## 4. Materials and Methods

### 4.1. Cultivation Condition and Selection of the Materials

We utilized two lines of *T. kok-saghyz*, namely T601 (approximately 4–5% natural rubber of root dry weight) and T1001 (approximately 6–8% of rubber content in the root) as previously characterized by Wu et al. [9]. All plant materials were cultivated in a tissue-culture-room (constant temperature: 25 ± 2 °C, light intensity: 12,000 Lx, relative humidity: 65%, light 16 h/dark 8 h).

### 4.2. Identification and Phylogenetic Analysis of GDS in T. kok-saghyz

Homology based screening of publicly available expression libraries led to the identification of the already reported *A. thaliana* geranyl diphosphate synthases (AtGDS1: AT2G34630; AtGDS2: AT4G36810). The *T. kok-saghyz* genome database (Accession Number: PRJCA005187) from the National Genomics Data Center to identify TkGDS [9]. Local BLASTp-2.9.0+ searches were conducted with an E-value threshold of 1 × 10^−30^. These identified TkGDS proteins were further analyzed to confirm the presence of the DDXXDD motifs, commonly referred to as FARM (first aspartate-rich motif, DDX_(2–4)_ DD) or SARM (second aspartate-rich motif, DDXXD), critical for prenyltransferase catalytic function [16,42]. This analysis identified seven putative members of the TkGDS family. To provide a robust evolutionary and functional context for the TkGDS family, we selected GDS proteins from representative species: *H. brasiliensis* (the primary commercial source of natural rubber), *A. thaliana* (a model plant with a comprehensively annotated genome), and *E. ulmoides* (another rubber-producing plant that independently evolved this trait). A phylogenetic tree was then constructed using the Neighbor-Joining (NJ) method in MEGA 10 software [43], enabled the assessment of evolutionary relationships among GDS proteins from *A. thaliana*, *E. ulmoides*, *H. brasiliensis* and *T. kok-saghyz*. The SOPMA and SWISS-MODEL programs were employed to predict secondary and three-dimensional structures, respectively. To investigate the evolutionary dynamics of this gene family, *TkGDS* sequences were analyzed to identify duplication events, and intra-species synteny maps were constructed. In addition, to explore the evolutionary conservation and divergence of GDSs across species, collinearity analyses were performed by integrating the genomic data of *A. thaliana* and *T. kok-saghyz* using TBtools-2.200 with default parameters, providing a comprehensive view of the syntenic relationships between *AtGDS* and *TkGDS*.

### 4.3. Motif, Domain, and Gene Structural Analysis of GDS

Multiple sequence alignments of all GDS proteins were performed using BioEdit. Conserved domains were identified within the GDS proteins to ensure alignment accuracy and evaluate homology, confirming the presence of essential prenyltransferase active motifs [44]. Conserved motifs and organizational patterns were analyzed with the Multiple Expectation maximization for Motif Elicitation (MEME) suite, revealing structural and functional distinctions among family members. Additionally, domain analysis was conducted using NCBI Conserved Domain Database (CDD) batch search, with TkGDS protein sequences submitted for structural annotation [45]. The chromosomal locations of the *TkGDS* genes were determined based on the *T. kok-saghyz* genome assembly (Accession Number: PRJCA005187) and visualized using MapInspect 1.0. Subsequently, TBtools-2.200 was employed for comprehensive visualization of gene and domain structures as well as motif distribution [46].

### 4.4. Structural Prediction of TkGDS Proteins

Secondary structure prediction for TkGDS proteins was conducted via SOPMA, which estimates the proportions of α-helices, β-turns, extended strands and random coils based on sequence characteristics [47]. Homology modeling was performed in SWISS-MODEL to predict the three-dimensional structure of TkGDS, providing structural insights potentially linked to their biological function [48].

### 4.5. Protein Interaction Network Analysis

We employed the online prediction tool STRING, drawing on data from experimentally validated interactions, curated databases, and information on co-expression, gene fusion events, text-based analysis and occurrence patterns. After performing k-means clustering and a preliminary analysis on STRING, results were exported to Cytoscape 3.9.0 for further refinement and visualization [49].

### 4.6. Cis-Acting Regulatory Elements Analysis

Genomic data of *T. kok-saghyz* were processed with TBtools-2.200 to extract 1500 bp upstream region of *TkGDS* genes for promoter analysis. These promoter sequences were submitted to PlantCare for *cis*-acting regulatory elements prediction [50], with subsequent data organization and filtering facilitated by Linux. Results were visualized using R-4.4.2 to demonstrate the distribution of regulatory elements across *TkGDS* promoters.

### 4.7. Transient Transformation Assay

We selected the 35S::GFP vector tagged with green fluorescent protein (GFP) as the expression vector. Specific primers *TkGDS*-F/*TkGDS*-R were designed to amplify the coding sequence (CDS) of *TkGDS* genes and listed in Table S1 and to clone them in-frame into the vector, generating the recombinant plasmids 35S::TkGDS1-GFP and 35S::TkGDS7-GFP. The detailed construction strategy is described in Section 4.9. *Nicotiana benthamiana* was transformed with the recombinant plasmids to achieve optimal protein expression levels and precise fluorescence signal detection [51]. Following transformation, the *N. benthamiana* plants were incubated in darkness for 12 h to enhance transfection efficiency, then exposed to light for 48 h to induce protein expression for observation. Finally, confocal microscopy was employed to examine the localization of TkGDS proteins within *N. benthamiana* leaf tissues, ensuring accuracy and specificity in signal detection [52]. The control group was established using the original 35S::GFP plasmid. *N. benthamiana* was transformed with constructed *A. tumefaciens* strain served to verify that the observed fluorescence signal specificity stemmed from the expression of TkGDS proteins in a single independent leaf. the use of appropriate fluorescent protein tags, the Agrobacterium-mediated transient expression in tobacco leaves, an OD600 of 1.0 for infiltration, a dark incubation period of 12–24 h post-infiltration, and observation using a Leica TCS SP8 confocal microscope. A schematic diagram of the expression cassette is provided in Supplementary Figure S6.

### 4.8. Expression Pattern Analysis of TkGDS Genes

Transcriptome data of *T. kok-saghyz* were accessed via local BLAST to extract FPKM expression profiles for each *TkGDS* gene in different tissues in line T601 as well as in line T1001 based on a previously published dataset (NCBI accession number: PRJNA1262623) [9], and expression profiles were visualized through a heatmap created using TBtools-2.200 software. For the further exploration of each gene expression level, qRT-PCR was carried out using the ChamQ Universal SYBR qRT-PCR Master Mix (Vazyme Innovation in Enzyme Technology, Nanjing, China) on a StepOne PCR system running StepOne Software v2.2.3. Primer sequences used for qRT-PCR are provided in Table S2. The *β-actin* gene (GenBank Accession No. DY824357) served as the internal reference [11]. Expression levels of selected *TkGDS* genes were quantified using the 2^−ΔΔCt^ method [53], and all experiments were performed with three independent biological replicates. Statistical significance of differences in qPCR data was determined by one-way analysis of variance (ANOVA) followed by Tukey’s honest significant difference (HSD) post hoc test, with *p* < 0.05 considered significant. Analyses were performed using Graphpad prism 10 software.

### 4.9. Construction of Recombinant Plasmids for Overexpression

Total RNA was extracted from the roots of *T. kok-saghyz* line T601 using an RNA extraction kit (Tsingke Biotechnology, Beijing, China). Approximately 2 μg of total RNA was reverse-transcribed into first-strand cDNA using the FastQuant RT Kit (Vazyme, Nanjing, China), which served as the template for *TkGDS* gene cloning. Gene-specific primers containing *Kpn*I and *Bam*HI restriction sites were designed to amplify the coding sequences (CDS) of *TkGDS1* and *TkGDS7*. The PCR products were digested and ligated into the pBI121-GFP expression vector to generate recombinant plasmids.

The resulting overexpression constructs, designated 35S::TkGDS1-GFP and 35S::TkGDS7-GFP, contained the respective *TkGDS* CDS (without stop codons) fused in-frame to the N-terminus of the GFP reporter gene, all under the control of the constitutive CaMV 35S promoter. A schematic diagram of the T-DNA region of the final vector is provided in Figure S6.

### 4.10. Transformation and Molecular Identification

Root explants from the T601 line of *T. kok-saghyz* were used for transformation. The recombinant plasmids were introduced into *A. rhizogenes* K599 competent cells. Transformation of *T. kok-saghyz* leaf explants utilized the CDB (Cut-Dip-Budding) method [9,54]. Briefly, about 3-week-old seedlings were cut and the wound sites were inoculated with *A. rhizogenes* K599 suspension (OD_600_ ≈ 1.0). Explants were co-cultivated with Agrobacterium to regenerate transgenic shoots. Photographs of the regenerating transgenic shoots are provided in Figure S7. After approximately one month of growth, genomic DNA was extracted from putative transgenic plants. Transgenic plants were identified by PCR using a primer pair that included a gene-specific forward primer and a reverse primer binding to the pBI121 plasmid backbone (pBI121-backbone-R, see Table S3). This strategy confirms the successful integration of the entire T-DNA expression cassette into the plant genome.

### 4.11. Analysis of TkGDS Genes in Overexpression Materials

Total RNA was isolated from the leaves of one-month-old seedlings of overexpression materials for qRT-PCR as mentioned above, which was conducted using ChamQ Universal SYBR qPCR Master Mix (Vazyme Innovation in Enzyme Technology, Nanjing, China), employing the StepOne Software v2.2.3 PCR System. Primers were designed based on *TkGDS* genes (Table S4). Each sample was run in triplicate, and the *β-actin* gene from *T. kok-saghyz* was employed as internal reference gene [11], data were normalized using the 2^−ΔΔCt^ method for relative quantification.

### 4.12. Quantification of Natural Rubber Content in T. kok-saghyz Roots

Fresh *T. kok-saghyz* roots were cleaned and dried to remove surface soil and moisture, and then *T. kok-saghyz* roots around 0.5 g of dried weight was incubated in a water bath for 1 h using 3% KOH, the mixture was stirred magnetically for 30 min. Rubber particles were manually collected, then washed with ddH_2_O, dehydrated, and weighed, as described [55]. Each quantification was performed with three independent biological replicates. The rubber content was calculated as a percentage of the root dry weight.

### 4.13. Morphology of T. kok-saghyz Photosynthesis

We employed a photosynthesis apparatus (Top Cloud-Agri Technology, Hangzhou, China) to determine photosynthetic parameters, which mainly involved net photosynthetic rate (PN), transpiration rate (TR), stomatal conductance (GS), intercellular CO_2_ concentration (CI), water use efficiency (WUE), and photosynthetically active radiation (PAR), for overexpressed plant materials. Each line was set up in triplicate to ensure statistical reliability.

### 4.14. Statistical Analysis

Data are presented as means ± SE from three independent biological replicates for any group unless otherwise noted. Statistical significance was assessed by one-way ANOVA with Tukey post hoc test (* *p* < 0.05, ** *p* < 0.01). Analyses were performed using SPSS (version 26.0), and graphs were created with Graphpad Prism 10.

## 5. Conclusions

Our results are consistent with the notion that the structural and functional diversification of *GDS* genes in *T. kok-saghyz* may have been driven by evolutionary pressures, suggesting potential dual roles for these proteins in both natural rubber biosynthesis and photosynthetic efficiency. Through comprehensive functional characterization of seven GDS members, we classified them into two evolutionarily distinct clades based on conserved motifs and structural domains. These proteins likely form homo- or heterodimeric complexes, consistent with findings in *H. brasiliensis* and *A. thaliana*. Chloroplast localization supports their involvement in plastidial isoprenoid metabolism. Promoter analysis revealed MeJA-responsive elements, and transcriptomic profiling identified *TkGDS1* and *TkGDS7* as potential key regulators, exhibiting strong expression and responsiveness to genotype and tissue types. Overexpression of *TkGDS1* and *TkGDS7* significantly increased rubber content (up to 6.08%) and photosynthetic efficiency, suggesting a compelling association whereby enhanced photosynthetic performance may contribute to increased carbon availability for rubber biosynthesis. Collectively, this study elucidates structural divergence and functional specialization of the *TkGDS* gene family. Specifically, *TkGDS1* and *TkGDS7* are strongly associated with enhanced chloroplast activity and increased rubber biosynthetic flux, establishing them as promising targets for efforts to engineer rubber yield in temperate-adapted crops in *T. kok-saghyz*.

## Figures and Tables

**Table 1:** Information about GDS family in *T. kok-saghyz*.

Gene	Gene ID	ORF/bp	PI	MW/ kDa	Size/ aa	Subcellular Localization *
*TkGDS1*	TkA06G134330	812	5.14	29.30	268	Chloroplast
*TkGDS2*	TkA06G097970	799	6.01	28.53	264	Chloroplast
*TkGDS3*	TkA07G129020	975	5.54	35.03	321	Chloroplast
*TkGDS4*	TkA07G129470	1269	6.33	46.38	419	Chloroplast
*TkGDS5*	TkA07G129240	1045	5.67	37.88	345	Chloroplast & Mitochondrial
*TkGDS6*	TkA07G129530	1272	6.08	46.30	419	Chloroplast
*TkGDS7*	TkA07G403060	1102	5.16	38.82	364	Chloroplast

*: Subcellular localization was confirmed by GFP fusion fluorescence assay. Experimental evidence is provided in 2.5. Subcellular Localization of TkGDS.

**Table 2:** Secondary structural prediction of TkGDS proteins.

Proteins	α-Helix (%)	β-Turn (%)	Extended Strand (%)	Random Coil (%)	Structure
TkGDS1	63.26	6.44	4.92	25.38	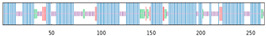
TkGDS2	61.57	7.09	5.97	25.37	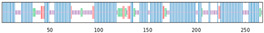
TkGDS3	63.64	5.67	5.39	25.30	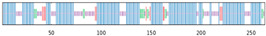
TkGDS4	66.67	5.22	4.35	23.77	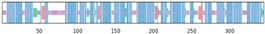
TkGDS5	53.94	4.06	5.73	36.28	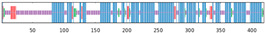
TkGDS6	62.31	6.54	5.61	25.55	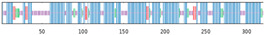
TkGDS7	59.07	5.49	7.42	28.02	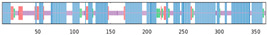

Color scheme: α-helix (blue), random coil (purple), extended strand (red), β-turn (green).

## Data Availability

The original contributions presented in the study are included in the article/Supplementary Material, further inquiries can be directed to the corresponding author.

## Supplementary Materials

The following supporting information can be downloaded at https://www.mdpi.com/article/10.3390/plants14192980/s1, Figure S1: Chromosomal localization of the *TkGDS1*-*TkGDS7* genes; Figure S2: Collinearity relationships of *GDS* genes; Figure S3: Tertiary structure of the TkGDS1-TkGDS7 proteins; Figure S4: Protein–protein interaction network of TkGDS proteins; Figure S5: Molecular identification of transgenic *T. kok-saghyz* line. (a) Validation of the *TkGDS1* transgene integration in overexpression lines. (b) Validation of the *TkGDS7* transgene integration in overexpression lines; Figure S6: Plasmid maps of the 35S::TkGDS-GFP constructs. a Vector map of 35S::TkGDS1-GFP. b Vector map of 35S::TkGDS7-GFP. Key components of the T-DNA region are indicated; Figure S7: GFP fluorescence in transgenic shoots under UV illumination. Images were taken one weeks after transformation. a 35S::TkGDS1-GFP, b 35S::TkGDS7-GFP* expressing shoots. White arrows denote GFP-positive areas; Table S1: Primer used for subcellular location of *TkGDS* genes; Table S2: Relative expression analysis of *TkGDS* genes across different tissues in T601 and T1001 of *T. kok-saghyz*; Table S3: Primers used for the construction of overexpression vectors and molecular verification; Table S4: Primer sequences for relative expression of overexpression materials; Table S5: Differentially expressed *TkGDS* genes were identified by comparisons between the transcriptome data of LRY lines and HRY lines using FPKM values; Table S6: Correlation analysis of *TkGDS* genes to line factors by ANOVA; Table S7: Correlation analysis of *TkGDS* genes to tissue factors by ANOVA; Table S8: Relative expression analysis of *TkGDS1* in overexpressed lines using 2^−ΔΔCt^ method; Table S9: Relative expression analysis of *TkGDS7* in overexpressed lines using 2^−ΔΔCt^ method.

## Abbreviations

The following abbreviations are used in this manuscript:

MVA	Mevalonate
MeJA	Methyl jasmonate
qRT-PCR	Quantitative real-time polymerase chain reaction
GFP	Green fluorescent protein
CDB	Cut-dip-budding Cut-dip-budding
OE	Overexpression
PN	Net photosynthetic rate
TR	Transpiration rate
GS	Stomatal conductance
CI	Intercellular CO_2_ concentration
WUE	Water use efficiency
PAR	Photosynthetically active radiation
