# Supplementary material for: Geranyl Diphosphate Synthases GDS 1 and GDS7 Facilitate Natural Rubber Biosynthesis in Taraxacum kok-saghyz Roots
Source: Plants (Basel). 2025 Sep 26;14(19):2980. doi: 10.3390/plants14192980 (PMC12526415; doi:10.3390/plants14192980)
Supplement: Supplementary file 1 [file plants-14-02980-s001.zip › Supplementary figures.pdf]

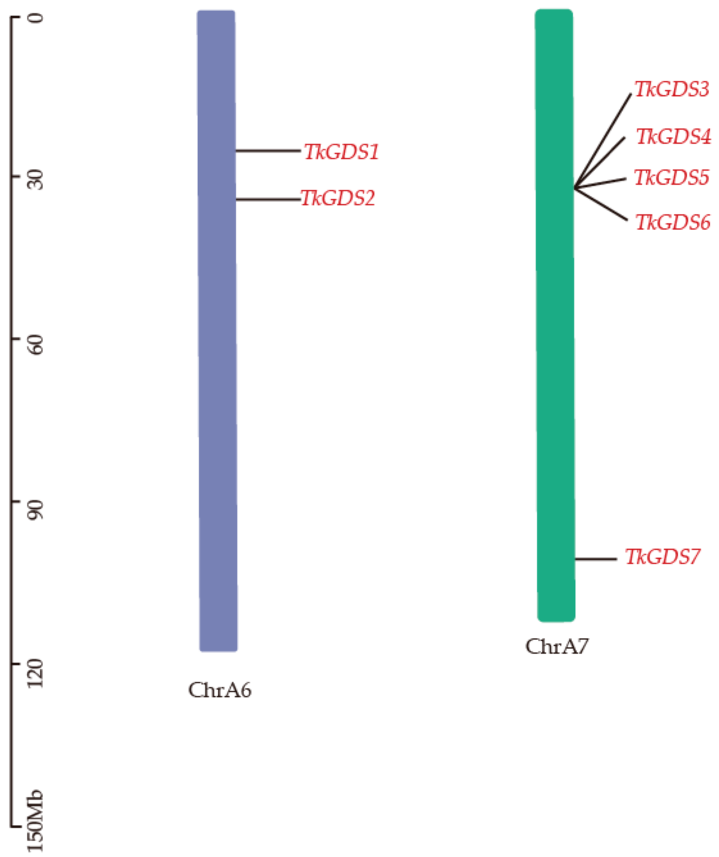

Figure S1. Chromosomal localization of the *TkGDS1*-*TkGDS7* genes.

a

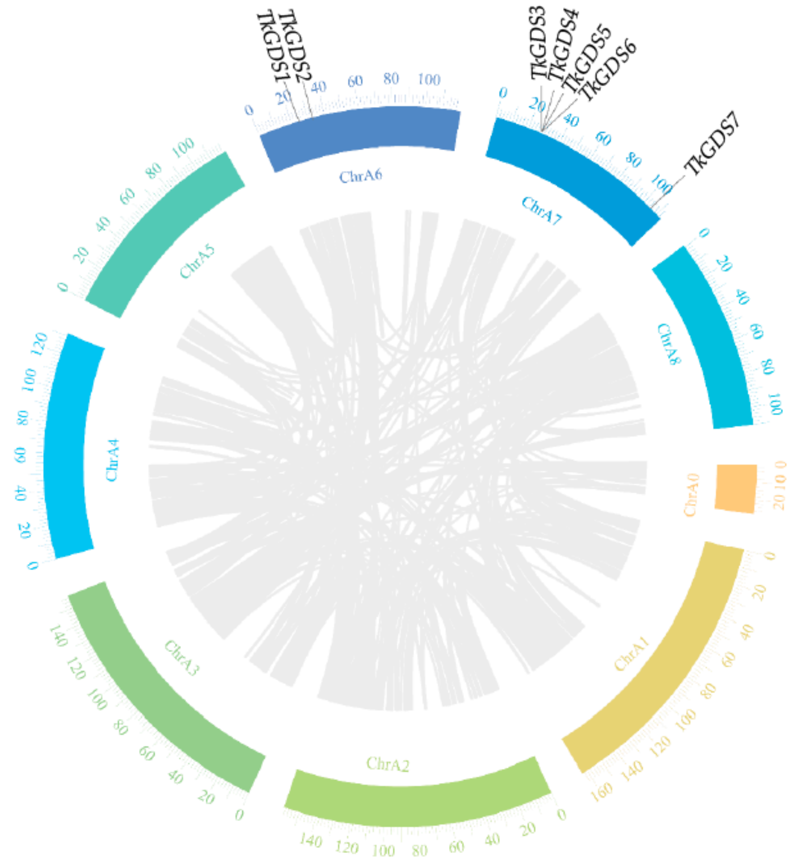

b

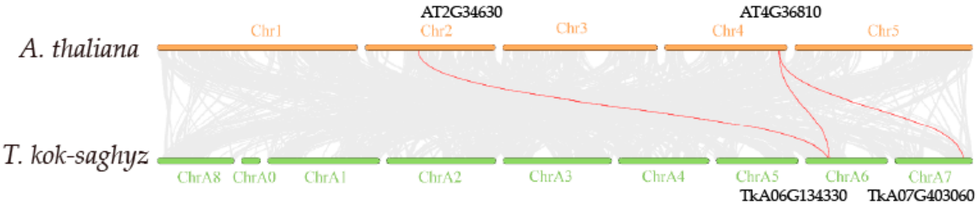

Figure S2. Collinearity relationships of *GDS* genes. (**a**) Intraspecies collinearity of *TkGDS* genes in *T. kok-saghyz*. Colored blocks/lines represent chromosomal clusters CluA4–CluA17, corresponding to *T. kok-saghyz* chromosomes ChrA0–ChrA8. Gray connecting lines indicate conserved syntenic relationships among *TkGDS* homologs. (**b**) Interspecies collinearity of GDS genes between *T. kok-saghyz* and *A. thaliana*. Syntenic regions are shown between *T. kok-saghyz* chromosomal clusters and *A. thaliana* chromosomes.

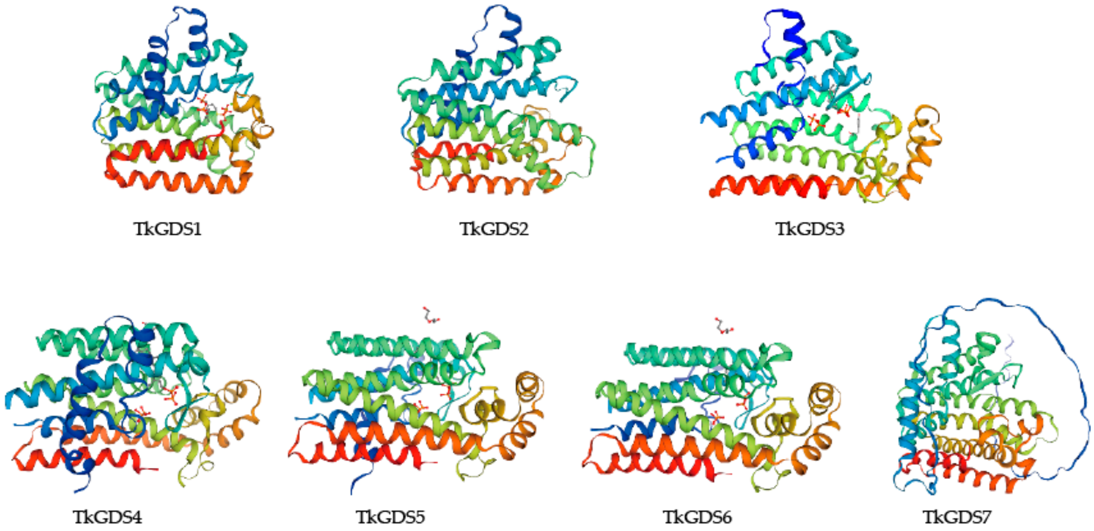

Figure S3. Tertiary structure of the TkGDS1-TkGDS7 proteins. Structures are shown in ribbon representation, with conserved functional domains highlighted in distinct colors.

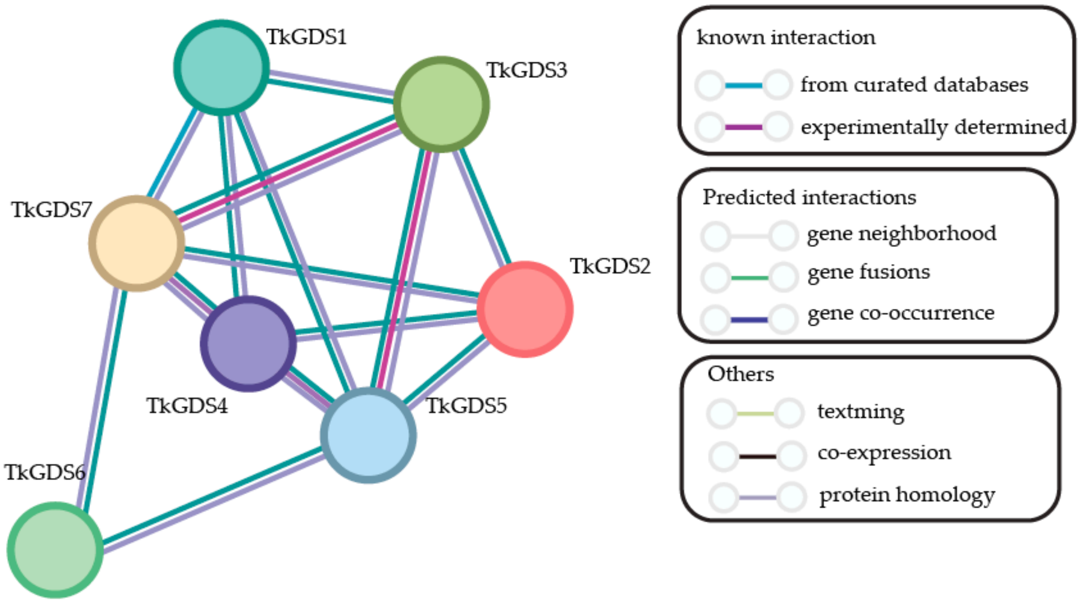

Figure S4. Protein-protein interaction network of TkGDS proteins. Predicted functional associations among GDS enzymes in *T. kok-saghyz*.

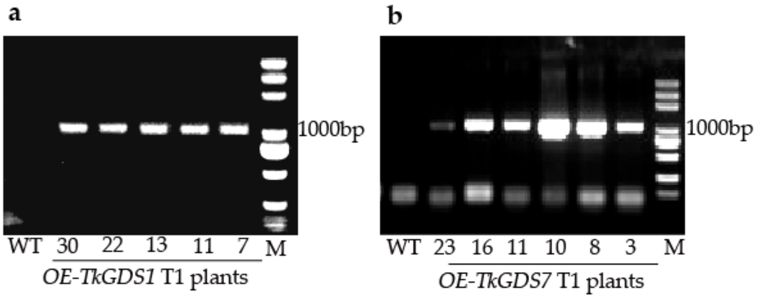

Figure S5. Molecular identification of transgenic *T. kok-saghyz* line. (a) Validation of the *TkGDS1* transgene integration in overexpression lines. (b) Validation of the *TkGDS7* transgene integration in overexpression lines.

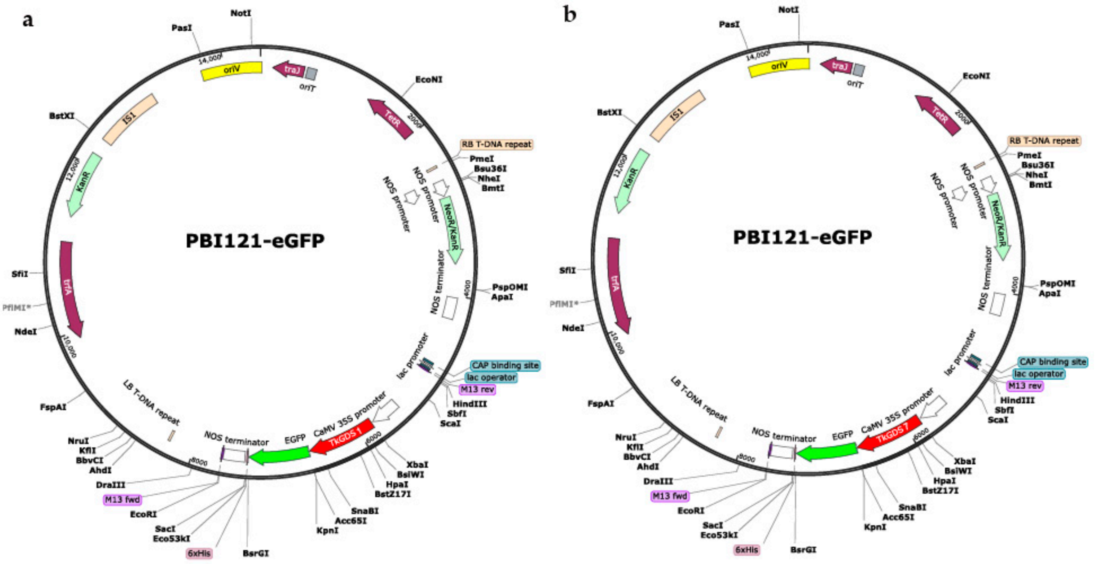

Figure S6. Plasmid maps of the 35S::TkGDS-GFP constructs. **a** Vector map of 35S::TkGDS1-GFP. **b** Vector map of 35S::TkGDS7-GFP. Key components of the T-DNA region are indicated.

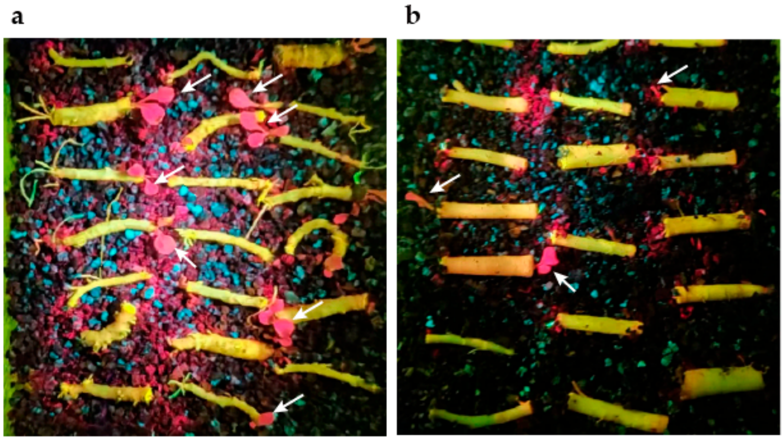

Figure S7. GFP fluorescence in transgenic shoots under UV illumination. Images were taken one weeks after transformation. **a** 35S::TkGDS1-GFP, **b** 35S::TkGDS7-GFP* expressing shoots. White arrows denote GFP-positive areas.
